# Characteristics and Outcomes of Physician-to-Physician Telephone Consultation Programs: Environmental Scan

**DOI:** 10.2196/17672

**Published:** 2021-02-23

**Authors:** Peter George Jaminal Tian, Jeffrey Richard Harris, Hadi Seikaly, Thane Chambers, Sara Alvarado, Dean Eurich

**Affiliations:** 1 Department of Family Medicine University of Alberta Edmonton, AB Canada; 2 Division of Otolaryngology Department of Surgery University of Alberta Edmonton, AB Canada; 3 University of Alberta Libraries University of Alberta Edmonton, AB Canada; 4 School of Public Health University of Alberta Edmonton, AB Canada

**Keywords:** telephone consultations, teleconsultations, remote consultations, telemedicine, eHealth, environmental scan

## Abstract

**Background:**

Telephone consultations between physicians provide quick access to medical advice, allowing patients to be cared for by calling physicians in their local settings.

**Objective:**

As part of a quality assurance study of a physician-to-physician consultation program in Alberta, Canada, this environmental scan aims to identify the characteristics and outcomes of physician-to-physician telephone consultation programs across several countries.

**Methods:**

We searched 7 databases to identify English publications in 2007-2017 describing physician-to-physician consultations using telephones as the main technology. To identify Canadian programs, the literature search was supplemented with an additional internet search.

**Results:**

The literature search yielded 2336 citations, of which 17 publications were included. Across 7 countries, 14 telephone consultation programs provided primary care providers with access to various specialists through hotlines, paging systems, or call centers. The programs reported on the avoidance of hospitalizations, emergency department visits and specialty visits, caller satisfaction with the telephone consultation, and cost avoidance.

**Conclusions:**

Telephone consultation programs between health care providers have facilitated access to specialist care and prevented acute care use.

## Introduction

Health care systems continuously evolve. At this point, a health care system’s efficiency is a measure of its ability to rapidly collect, store, and analyze information and make it accessible in real time to a wide range of health care providers to optimize patient care. A key component of these systems is the use of technology to allow health care providers to easily consult and securely share patient information with other providers. The World Health Organization (WHO) promotes this use of information communication technology (called eHealth) in support of health care services and training. In a 2016 survey, the WHO reported that 58% of responding member states had eHealth strategies, and 62% of member states had a consultation service using mobile information communication technology between health care practitioners or between health care practitioners and patients [1].

This ubiquitous use of technology in health care is reflected in published literature, and the benefits of these systems to care processes have been well documented. A systematic review by Deldar et al [2] found 174 publications examining the role of teleconsultations. Another systematic review by Saliba et al [3] identified 94 studies evaluating the facilitators and barriers of various telemedicine services. The delivery of such eHealth solutions is substantial. Teleconsultations in dermatology [4] and psychiatry [5], for example, may come in different modalities and be provided using videoconferencing and store-and-forward systems (ie, sending images and text information). Teleradiology, which has been around for decades, allows for the transmission, storage, and retrieval of images between radiologists and other professionals [6]. Many other technologies may be used to serve as a means of accessible electronic medical records, mobile telephone symptom recordings, and dedicated support lines, as used, for example, in palliative care [7].

In Alberta, Canada, physicians have access to telephone consultations with specialists through a 24/7 call center called RAAPID (Referral, Access, Advice, Placement, Information, and Destination). RAAPID ensures that physicians have quick access to other physicians (often specialists) for advice, allowing patients to be cared for by the calling physician in their local setting. However, if patients require transfer to other institutions for care, RAAPID also facilitates these transfers [8]. One component of the RAAPID system that has been increasingly utilized is telephone consultation with Otolaryngology–Head and Neck Surgery (OHNS). As part of a quality assurance study to evaluate RAAPID’s telephone consults with OHNS, we conducted an environmental scan of similar programs in other parts of the world, searching for program characteristics and outcomes associated with similar physician-to-physician telephone consultation programs.

## Methods

We used a combination of formal literature searches and internet searches based on methods adapted for the conduct of an environmental scan [9]. Other published environmental scans have also used internet searches for gathering data [10,11]. Since the RAAPID program provides consultation using only phones between physicians, we limited our search to programs that included physician-to-physician consultation with telephone as the main technology.

An information technologist (author TC) performed the literature search. The search was done on the following databases: Ovid MEDLINE(R), Embase, Cochrane Database of Systematic Reviews, Cochrane Central Register of Controlled Trials, NHS Economic Evaluation Database, CINAHL, and Web of Science Core Collection. The search was limited to English publications in the 10-year range of 2007-2017 to ensure that identified articles reflected more contemporary practice in the field. To identify Canadian programs, the literature search was supplemented with an additional internet search (by author PT) using the following search terms in Google: (Physician or Doctor) AND (Telephone Consultation or Phone Consultation). Then we reviewed potentially relevant search results and websites. The internet search results were limited to the first 10 search result pages (~100 results).

Three authors (PT, TC, SA) screened the search results for programs providing access to specialists through telephone consultations. For the results from the literature search, a 2-step screening was performed: title-abstract screening and full-text review to identify relevant studies. The title-abstract screening was performed independently by 2 authors (PT and TC; PT and SA). Then, full-text screening was independently reviewed by 2 authors (PT and SA). Disagreements in the screening decisions were resolved by discussion. Only consultation programs that used the telephone as the main technology were included, as well as studies that used telephones for initial consultations before using other technologies at a later time. However, consultation programs that used the telephone in combination with other technologies (such as fax, video platform, electronic communication, mobile messaging, and web-based platforms) were excluded.

One author (PT) extracted the data, and the data were verified by a second author (SA). The following data were extracted from the publications and the internet search results: the name of the program, the country within which the program operates, a description of the program, who the program is available to, the scheduled times and specialty areas in which the service is available, reported measures (ie, the volume of calls, response times), disposition after consultation (ie, sent home, sent to the emergency department or hospital, elective consultation in a specialty clinic), satisfaction with the calls, and potential costs or cost avoidance. For the supplemental internet search, one author (PT) reviewed the search results and extracted the data.

## Results

### Literature and Internet Search

**Figure 1:**
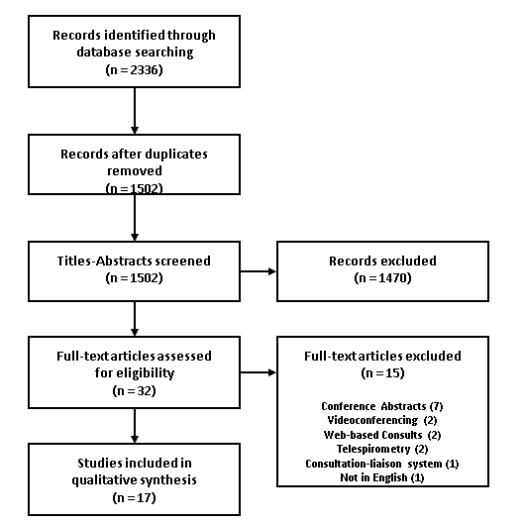
Flow chart of the literature review process.

The literature search yielded 2336 citations, of which 17 publications were identified and included (Figure 1). These 17 publications described 14 telephone consultation programs: 4 programs in the United States, 3 programs each in Canada and France, and 1 program each in Australia, the Netherlands, the United Kingdom, and Italy (Table 1). The sample sizes of the included consultation programs ranged from 19 to 4436. In addition to these publications, the internet search yielded 17 webpages linked to 13 Canadian telephone consultation programs (Table 2).

**Table 1:** Characteristics of telephone consultation programs (n=14).

Publication (first author, publication year, country)	Program name or description	Access schedule	Physician seeking consultation	Physician providing consultation (number of calls and duration)	Post-call patient disposition
Bal, 2011, France.	Hotline on a dedicated cellular telephone	24 hours per day, 7 days per week	General practitioner	Infectious disease resident and specialist (284 calls in 6 months)	—^a^
Clark, 2015, Canada.	Randomized trial comparing usual care with telephone consult	—	Primary care physician	Pain specialist at 0 months, 3 months, and 6 months (n= 41)	—
Hilt, 2013, US.	The Partnership Access Line (toll-free number)	8 AM-5 PM, Monday-Friday	Primary care provider	Child and adolescent psychiatrist (2285 calls in 37 months)	—
Hobbs, 2014, US; Sarvet, 2011, US; Sarvet, 2010, US; Straus, 2014, US.	Massachusetts Child Psychiatry Access Hotline (answered by the care coordinator and routed to an appropriate team member)	Business hours, Monday-Friday	Pediatric primary care clinician (eg, pediatrician, family practice physician, nurse practitioner)	Child psychiatrist, family psychotherapist, care coordinator (4436 calls in 1 year)	24% (1974/8223) of consults resulted in the primary care clinician maintaining primary clinical responsibility
Lear, 2010, Canada.	Rapid Access to Cardiology Expertise (pilot project for Wilson, 2016, Canada, listed below; paging system which initiates a call)	Business day	Family physician	Cardiologist (118 calls in 7 month); the cardiologist calls the paging family physician	17.8% of consults resulted in further consultation with the cardiologist
Linklater, 2009, UK.	Telephone advice line	24 hours per day, 7 days per week	Primary care clinician (eg, general practitioner, hospital doctor, hospital/community nurse, patient or carer)	Consultant or specialist registrar in palliative medicine (1146 calls in 6 years and 1 month)	—
Marquet, 2013, France.	National network of infectious disease experts	—	Community and health care professional	Infectious disease specialist (323 calls in 5 days)	6% of consults led to infectious disease consultation; 5.5% led to hospitalization
Salles, 2014, France.	Hotline	9 AM-7 PM, Monday-Friday	General practitioner	Geriatrician (714 calls in 16 months)	38.3% of consults resulted in advice; 5.3% resulted in geriatric consultation; 9.2% resulted in a hospital day visit; 42.9% resulted in hospitalization in the geriatrics ward; 4.3% resulted in direct admission to the emergency department
Sankaranarayanan, 2010, Australia.	Telephone line	12 PM-1 PM, Monday-Friday	General practitioner	Psychiatrist (19 discussions in 3 months)	—
van Heest, 2008, Netherlands.	Telephone line	24 hours per day, 7 days per week	Health care provider (eg, general practitioner, nurse, pharmacist, other health care provider)	General practitioner adviser in palliative care on treating nausea and vomiting (572 consultations in 1 year)	—
Waldura, 2013, US.	HIV Warmline	9 AM-8 PM	Primary care clinician (eg, physician, other health care provider)	HIV specialist (eg, physician, pharmacist)	—
Wegner, 2008, US.	Telephone line	—	Primary care physician	Pediatric subspecialist (306 consults in 8 months)	32% of consults avoided a PS^b^ visit; 11% avoided a hospital transfer; 5% avoided a hospital admission; 5% avoided an emergency department visit
Wilson, 2016, Canada.	Rapid Access to Consultative Expertise (hotline that automatically routes to a specialist’s pager/mobile phone)	8 AM - 5 PM, Monday-Friday	Family physician or nurse practitioner	Various specialists (a subset of 2000 calls in 2 years)	60% of consults prevented a face-to-face consultation; 32% prevented an emergency department visit
Zanaboni, 2009, Italy.	Telephone calls routed through a service center; for specific consultations, callers were invited to use biomedical devices for specific consultations	—	General practitioner	Cardiologist, dermatologist, or diabetologist (927 cardiology calls in 25 months)	8% of consults resulted in an emergency department visit or hospitalization; 1% resulted in an in-clinic visit; 77% avoided an emergency department visit, hospitalization, or an in-clinic consult

^a^—: not available.

^b^PS: pediatric subspecialist.

**Table 2:** Characteristics of Canadian physician-to-physician telephone consultation programs yielded from an internet search (n=13).

Program Name	Province	Program description	Physician seeking consultation	Physician providing consultation
Cancer Line	Alberta	Assists with cancer-related questions	Physician or other health care provider	Medical or radiation oncologist, or expert oncology nurse
Orthopedic Consult Line	Alberta (Edmonton)	—^a^	—	—
PaedLink Telephone Consultation Service^b^	Alberta (Calgary)	Single access number; accessible from 8 AM-8 PM, Monday-Sunday	—	—
Referral, Access, Advice, Placement, Information, and Destination (RAAPID)	Alberta	Hotline accessible 24 hours per day, 7 days per week	Physician	Multiple specialists
Specialist LINK^b^	Alberta (Calgary)	Telephone advice for nonurgent cases; accessible from 8 AM-5 PM, Monday-Friday, except on statutory holidays	Physician, nurse practitioner, midwife, pediatrician	Multiple specialists
Rapid Access to Consultative Expertise	British Columbia, Yukon	Accessible from 8 AM-5 PM, Monday-Friday	Physician, nurse practitioner	Multiple specialists
Rapid Access to Consultative Expertise	Manitoba	—	—	—
Med-Response	North West Territories	—	—	—
CritiCall-Ontario	Ontario	—	—	—
Ontario Shores	Ontario	Telephone advice; online booking	Family physician, nurse practitioner	Psychiatrist
Leveraging Immediate Non-urgent Knowledge (LINK)	Saskatchewan	Physician-to-physician telephone consultation service for nonurgent conditions; accessible from 8 AM-5 PM, Monday-Friday	—	Multiple specialists
Acute Care Access Line (ACAL)	Saskatchewan	Urgent calls; complementary to LINK service	—	—
Bedline	Saskatchewan	—	—	—

^a^—: not available.

^b^Eligible for continuing medical education (CME) credits.

### Telephone Consultation Process

**Figure 2:**
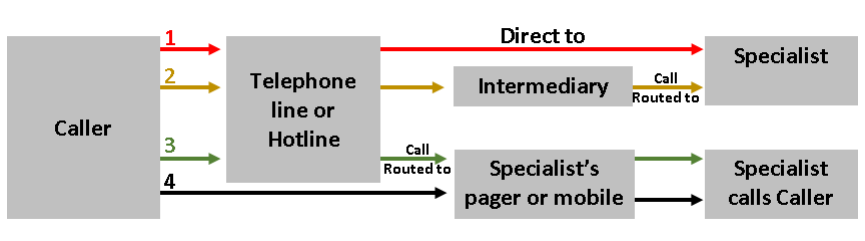
Various telephone consultation processes.

The process starts with the provision of a telephone line. Some publications used the term *hotline*; however, since no reported definitions of a hotline differentiate it from a regular telephone line, we extracted the terms as published. Of the 17 included studies, 10 reported a program in which the call connects and is answered directly by the specialist [12-21], 4 indicated programs in which the call is answered by an intermediary who then routes the call to the specialist [22-25], and 2 indicated that the call is routed to a specialist’s pager or mobile messaging service, requiring the specialist to respond with a call to the physician (Figure 2) [26,27].

### Accessibility

The accessibility of telephone consultations varied. Some were available 24 hours a day, 7 days a week [12,15]. Others were only available during business hours or extended business hours, Mondays to Fridays [13,16,22,26,27]. One program was limited to 1 hour a day, 5 days a week [14].

Response time between the programs also varied. Wilson [27] reported that 78% of calls were responded to within 10 minutes. Lear [26] reported that 81.4% of calls were returned within 1 hour or less. These 2 studies used a system where providers paged specialists who called them back. Marquet [28] reported that 19.8% of calls were answered immediately.

### Callers

Most of the programs were geared toward family physicians, general practitioners, or primary care providers [12-15,19,24,26,27]. In addition to physician callers, some programs also had nonphysician callers: nurse practitioners, pharmacists, and other professionals [15,16,27,28]. Other programs were highly restricted, such as the Massachusetts Child Psychiatry Access, which was limited to pediatric primary care clinicians like pediatricians, nurse practitioners, and family physicians [21-23].

### Consulted Specialists

The different programs offered consults with different specialties. The Rapid Access to Consultative Expertise (RACE) in British Columbia offered access to a wide number of specialists [27]. However, the majority of the studies reported consults with only certain physician specialists, namely, psychiatrists [14,22], infectious disease specialists [12,28], geriatricians [13], pediatricians [17,22], and cardiologists [24,26]. One reported access to general practitioners who served as advisers [15]. Others provided access to nonphysician members of the team, such as pharmacists, psychotherapists, or care coordinators [16,22].

### Patient Disposition

Only 6 (35%) of the 17 publications assessed any type of disposition within the program. With respect to patient and medical advice, only 1 publication explicitly noted this feature. Salles [13] reported that 38.3% resulted in advice. Although Hobbs [22] did not report this, another publication reported that in the Massachusetts Child Psychiatry Access program, 24% of consults resulted in the primary care clinician maintaining primary care responsibility [21]. Several publications noted that additional consultation occurred as a result of the call. Lear [26] reported that 17.8% resulted in further consultation with the cardiologist. Marquet [28] reported that 6% led to infectious disease consultation. Salles [13] reported that 5.3% resulted in geriatric consultation. Wegner [17] reported that 32% avoided pediatric subspecialists’ visits. Wilson [27] reported that 60% prevented a face-to-face consultation. Several publications (5/17, 29%) assessed emergency department visits or hospitalization as an outcome of the program [13,17,24,27,28]. However, the studies differed in how the patient dispositions were reported. Zanaboni [24] reported that 8% resulted in emergency department visits or hospitalization. Marquet [28] reported that 5.5% led to hospitalization. Salles [13] reported that 9.2% resulted in a hospital day visit, 42.9% resulted in hospitalization or a visit to a geriatrics ward, and 4.3% in a direct emergency department admission [13]. Conversely, several publications noted large effects with respect to avoidance of emergency department visits or hospitalizations. Indeed, Zanaboni [24] reported that 77% of calls avoided emergency department visits or hospitalizations. Wilson [27] reported that 32% avoided emergency department visits, while Wegner [17] reported that 5% avoided emergency department visits and 5% avoided hospital admissions.

### Cost Avoidance

Cost avoidance from the telephone consultations was reported in 3 of 17 publications (18%) and varied depending on how the studies determined cost avoidance. Wegner [17] reported cost savings of $477,274 for 306 consults over 8 months. These cost savings included subspecialist visit and telephone consultation costs, as well as potential costs from avoided hospitalizations. Wilson [27] reported cost savings of $9005 for 148 calls. Zanaboni [24] reported €20,472 of direct savings for in-clinic visits for 927 calls [24].

### Satisfaction With Calls

Of the 17 publications, 9 (53%) determined satisfaction levels after the telephone consultations. Physicians rated the telephone consultations positively, ranging from 80%-100% [12,24,26]. The ratings comprised satisfaction with the specialist’s recommendations, whether issues were addressed adequately, and improved confidence in managing patients. Also, compliance to recommendations were rated high, ranging from 90%-93% [12,15,24].

## Discussion

Our environmental scan identified 17 studies on telephone consultation programs between health care practitioners, and 13 programs across Canada. Programs were widely dispersed across a wide range of specialties and disease states. Overall, the most common model for accessing care was having the physician connect directly with the specialist or consultant rather than using a routing system or call-back procedure. The majority of programs were for physicians; however, many were supportive of calls from other members of the care team.

Interestingly, less than half of the publications evaluated outcomes related to patients’ dispositions or costs. In the few studies that evaluated health care utilization, all reported an avoidance in either emergency department visits or hospitalizations. As expected, this translated into major cost savings for the programs. However, it is relatively unclear what the overall net savings and costs of these programs were, as few (if any) analyses accounted for the input costs of operating and maintaining these programs. Indeed, British Columbia’s RACE program [27] has a low operating cost. It provides a hotline system that directly pages a specialist who, in turn, calls the referring physician. RACE reports a cost of only $120/month for the telephone system support and an administrative support cost for 1 day per month. Costs savings and some costs occurrence to the system would be expected; however, the benefits to patients in terms of timely medical advice and indirect cost savings (eg, travel to the emergency department, time away from work) would likely offset any cost occurrences. Coupled with reduced pressure on the emergency department and hospital system reported by the programs, the benefits are likely substantial.

RAAPID’s 24/7 call center shares some similarities to other telephone programs, but also notable differences. Unlike most programs that involve direct calling to specialists, physicians call a hotline and the call center connects them to specialists, if needed. The call center provides extensive support. At the consultation level, the call center triages the call to specialists, ascertains that the consultation occurred, and provides logistical support during and after the consultation. At a system level, the call center, when required, coordinates the transfer of patients to appropriate centers, with due consideration to bed management. However, this labor-intensive process impacts the operating cost (ie, the cost from 24/7 staffing).

Although more expensive to implement, the RAAPID program has previously reported that from November 2014 to October 2015, of 51,171 telephone consultations, 36% were not referred to the emergency department (29.1% resulted in the provision of advice and 6.9% were referred to a specialist clinic) [29]. This coincides with the figures observed in other programs reported in this paper. British Columbia’s RACE reported a 32% prevention rate in emergency department visits [27]. Wegner’s study [17] on pediatric subspecialist consultations reported that 52% avoided emergency department visits, specialty visits, and hospital transfers and admissions. Zanaboni’s study [24] on consultation calls to cardiology, dermatology, and diabetology reported that 77% avoided emergency department visits, hospitalizations, or in-clinic consults.

This environmental scan is the first narrative review of telephone consultation programs. We have reviewed the published literature and performed a supplemental internet search. However, the heterogeneity of programs and outcome measures limited comparison across programs. Moreover, limitations in resources have precluded a systematic review and a more extensive review of internet searches. The pervasive use of technology in health care consultations was evident in the literature search and internet search. The use of phones for consultations was minor compared to the use of more recent technologies like videoconferencing, mobile messaging, and other electronic and web-based platforms.

### Conclusion

Telephone consultation programs between health care providers have facilitated access to specialists. The programs have allowed primary care providers to retain the care for their patients while avoiding patient use of acute care resources. These telephone consultation programs, along with newer technologies, have increased the efficiency of health care.

## Abbreviations

OHNS: Otolaryngology–Head and Neck Surgery
RAAPID: Referral, Access, Advice, Placement, Information, and Destination
RACE: Rapid Access to Consultative Expertise
WHO: World Health Organization
